# Feasibility and Safety of Whole-Body Electromyostimulation in Frail Older People—A Pilot Trial

**DOI:** 10.3389/fphys.2022.856681

**Published:** 2022-06-24

**Authors:** Joerg Bloeckl, Sebastian Raps, Michael Weineck, Robert Kob, Thomas Bertsch, Wolfgang Kemmler, Daniel Schoene

**Affiliations:** ^1^ Institute of Medical Physics, Friedrich-Alexander University Erlangen-Nürnberg, Erlangen, Germany; ^2^ Institute for Biomedicine of Aging, Friedrich-Alexander University Erlangen-Nürnberg, Nuremberg, Germany; ^3^ Institute of Clinical Chemistry, Laboratory Medicine and Transfusion Medicine, Nuremberg General Hospital, Paracelsus Medical University, Nuremberg, Germany; ^4^ Institute of Radiology, University Hospital Erlangen, Friedrich-Alexander University Erlangen-Nürnberg, Erlangen, Germany; ^5^ Institute of Exercise and Public Health, University of Leipzig, Leipzig, Germany

**Keywords:** frailty, aged, functional capacity, safety, rhabdomyolysis, electric stimulation

## Abstract

Whole-body electromyostimulation (WB-EMS) induces high-intense stimuli to skeletal muscles with low strain on joints and the autonomic nervous system and may thus be suitable for frail, older people. However, if trained at very high intensities, WB-EMS may damage muscles and kidneys (rhabdomyolysis). This study aimed at investigating the feasibility, safety and preliminary efficacy of WB-EMS in frail, older people. Seven frail (81.3 ± 3.5 years), 11 robust (79.5 ± 3.6 years), 10 young (29.1 ± 6.4 years) participants completed an eight-week WB-EMS training (week 1–4: 1x/week; week 5–8: 1.5x/week) consisting of functional exercises addressing lower extremity strength and balance. Feasibility was assessed using recruitment, adherence, retention, and dropout rates. The satisfaction with WB-EMS was measured using the Physical Activity Enjoyment Scale for older adults (PACES-8). In week 1, 3, and 8 creatine kinase (CK) was assessed immediately before, 48 and 72 h after WB-EMS. Symptoms of rhabdomyolysis (muscle pain, muscle weakness, myoglobinuria) and adverse events were recorded. Functional capacity was assessed at baseline and after 8 weeks using the Short Physical Performance Battery (SPPB), Timed Up-and-Go Test (TUG), Choice Stepping Reaction Time Test (CSRT), 30-second Chair-Stand Test (30-STS), maximum isometric leg strength and handgrip strength. The recruitment rate of frail individuals was 46.2%, adherence 88.3% and the dropout rate 16.7%. All groups indicated a high satisfaction with WB-EMS. CK activity was more pronounced in young individuals with significant changes over time. Within older people CK increased borderline-significantly in the frail group from baseline to week 1 but not afterwards. In robust individuals CK increased significantly from baseline to week 1 and 3. No participant reached CK elevations close to the threshold of ≥5,000 U/l and no symptoms of rhabdomyolysis were observed. With the exception of the TUG (*p* = 0.173), frail individuals improved in all tests of functional capacity. Compared to the young and robust groups, frail individuals showed the greater improvements in the SPPB, handgrip strength, maximum isokinetic hip-/knee extension and flexion strength. WB-EMS is feasible for frail older people. There were no clinical signs of exertional rhabdomyolysis. WB-EMS proved to be sufficiently intense to induce meaningful changes in functional capacity with frail individuals showing greater improvements for several measures.

## 1 Introduction

Frailty is defined as “state of increased vulnerability to poor resolution of homoeostasis after a stressor event” (Clegg et al., 2013) and is associated with numerous adverse events, including falls, disability, hospitalization, loss of independence, and increased mortality (Vermeiren et al., 2016). Sarcopenia is a key determinant of physical frailty (Bernabei et al., 2014). During aging, complex mechanisms lead to a decrease in muscle quantity and quality (McGregor et al., 2014), affecting more pronounced the lower extremities (Amaral et al., 2014). In particular, atrophy, decline in number and size, and denervation of type II muscle fibres are associated with loss of functional capacity in old age, e.g., getting up from a chair or gait initiation (Lang et al., 2010). Exercise training is a key element in the prevention of sarcopenia and frailty as it has positive effects on different involved physiological systems (e.g., muscular system) and biological processes (e.g., inflammation) (Dent et al., 2018; Dent et al., 2019). Progressive resistance training and more specific, high-intensity (HIT) and power training are effective strategies to improve muscle mass, strength and functional capacity in older adults (Izquierdo et al., 2021).

Often however, frail older people suffer from functional limitations and multimorbidity (Vetrano et al., 2019), leaving them unable or unmotivated to engage in adequately intensive exercise programs. Here, whole-body electromyostimulation (WB-EMS) can induce training stimuli similar to HIT with lower strain on joints and the autonomic nervous system (Kemmler et al., 2015; Watanabe et al., 2019). WB-EMS is defined as “simultaneous application of electric stimuli via at least six current channels or participation of all major muscle groups, with a current impulse effective to trigger muscular adaptations” (Kemmler et al., 2020a). One characteristic of WB-EMS is the immediate recruitment of type II fibres, which have a higher excitation threshold than type I fibres (Hennemann’s size principle) (Maffiuletti, 2010).

WB-EMS has demonstrated to be effective in untrained and morphometrically sarcopenic (excluding the functional component) older people (Kemmler et al., 2016a; Kemmler et al., 2018; Kemmler et al., 2021). Limited evidence shows effects on muscle mass, handgrip and lower extremity strength, gait speed, cardiometabolic and anthropometric parameters (Kemmler et al., 2018; Kemmler et al., 2016a; Kemmler et al., 2017; von Stengel et al., 2015) as well as reductions of low back pain intensity and frequency (Weissenfels et al., 2017). However, participants in previous WB-EMS studies were overwhelmingly robust without functional impairments. To date, there is little evidence to support the efficacy of WB-EMS on important outcomes in frail, geriatric cohorts, such as functional capacity, balance, falls, and continence.

In addition, little is known about the safety of WB-EMS in frail older people. Especially in initial training periods, WB-EMS poses the risk of inducing severe muscle damage due to muscular overload (Stöllberger and Finsterer, 2019). This is likely due to the distinctive and immediate type II fibres recruitment and subsequent increased speed and severity of muscle damage (Nosaka et al., 2011). Cases of rhabdomyolysis, a severe and potentially life-threatening form of muscle damage, accompanied by a massive release of muscle proteins into the blood, which bears the risk of acute renal failure have been reported in younger adults with pre-existing unknown myopathies and elite athletes, when added to the usual training regimen (Stöllberger and Finsterer, 2019). Symptomatically, a triad of muscle pain, muscle weakness, and dark-coloured urine (myoglobinuria) can be prevalent, although in 50% of cases merely nonspecific symptoms occur, such as fever, tachycardia, nausea, vomiting, or general malaise (Cervellin et al., 2017). In addition, there is a large increase in muscle-specific serum biomarkers with elevations of CK by 117-fold (28,545 ± 33,611 U/l) and of myoglobin by 40-fold after WB-EMS with intentionally supramaximal workload (Teschler et al., 2016).

In the non-medical setting, older people with comorbidities (e.g., chronic kidney disease), usually have to be excluded from WB-EMS due to contraindications (Kemmler et al., 2019). Further, it remains unclear whether WB-EMS is accepted by frail older people, e.g., because of discomfort of the electrical stimuli (O’Connor et al., 2018). Geriatric syndromes like immobility, instability, incontinence, and cognitive impairment (Inouye et al., 2007), may also prevent the feasible administration of WB-EMS in this population.

To overcome some of these limitations and in preparation of a randomised controlled trial, we conducted a pilot study. The objectives were to evaluate the:

1) Feasibility of WB-EMS in frail older adults living in assisted living facilities based on recruitment, adherence, retention and dropout rates, as well as the satisfaction with and acceptability of WB-EMS in this population.
2) Safety of WB-EMS by observing the incidence of symptoms of exertional rhabdomyolysis via close monitoring of CK and clinical symptoms.
3) Preliminary efficacy of 8 weeks of WB-EMS in robust and frail older adults as well as a young comparison group in strength, functional capacity and mobility. This was also of interest to demonstrate that an adapted WB-EMS protocol would not lead to a lack of efficacy. In addition, the effect of WB-EMS on the Short Physical Performance Battery (SPPB), a test battery of lower extremity function, was examined to enable a realistic sample size calculation.

## 2 Materials and Methods

This study was carried out between July and December 2020 by the Institutes of Medical Physics and Biomedicine of Aging, Friedrich-Alexander University Erlangen-Nürnberg (FAU), Germany. The Ethics Committee of the Medical Faculty of the FAU approved the study (number 390_19B). It was conducted in accordance with the Declaration of Helsinki. The study report adheres to the Consolidated Standards of Reporting Trials (CONSORT) recommendations and their supplements for non-randomized pilot studies (Eldridge et al., 2016; Lancaster and Thabane, 2019). The trial was registered with clinicaltrials.gov (NCT04495647).

### 2.1 Trial Design

This study is a quasi-experimental trial in a pretest-posttest design with three study arms consisting of 1) a frail group (FG) and 2) a robust group (RG) of older people and 3) a young comparison group (YG). This approach allowed to determine the effects of age and frailty on WB-EMS’ feasibility, safety and efficacy.

### 2.2 Participants and Setting

For pragmatic reasons, it was decided to recruit *n* = 10 participants per group. Due to the Covid-19 pandemic, recruitment was halted after seven frail older people.

Inclusion criteria for the FG and RG were: age ≥75 years, no previous EMS exposure, and the ability to walk 4 m (with walking aid but without personal assistance). Inclusion criteria for the YG were: age < 40 years, healthy and no previous EMS exposure. Exclusion criteria for all groups were: severe visual or hearing impairments, major cognitive impairment (MMSE <10), medications with muscle-anabolic effects, medical conditions affecting trainability of muscles (e.g., Myasthenia gravis), surgery within the past 2 months (in the EMS stimulation area), history of rhabdomyolysis, general contraindications for physical training, medical conditions affecting sensation of electrical stimuli (e.g., severe polyneuropathy), acute or untreated abdominal wall or inguinal hernia, chronic kidney disease (CKD) stage 4 (GFR < 30 ml/min/1.73 m^2^) and electronic implants (e.g., pacemaker, defibrillator).

Older participants were classified into “robust” and “frail” using the Tilburg Frailty Indicator (TFI) (Freitag et al., 2016) with a cut-off score of ≥5 out of 15 points (Gobbens et al., 2020). The TFI is a validated self-report questionnaire that assesses three domains based on a biopsychosocial frailty model (Gobbens et al., 2010). It has good internal consistency (Cronbach’s *α*= 0.73), good test-retest reliability (ICC = 0.79), and high predictive validity for disability and care dependency (AUC >0.8) (Gobbens et al., 2010; Gobbens et al., 2020).

First, younger participants were recruited among investigators’ colleagues and university students via verbal information and E-mails. Second, robust older participants were recruited via the participant database of the Franconian Osteopenia and Sarcopenia Trial (FrOST), a study of 43 men (78.5 ± 4.2 years) who had completed an HIT over a period of 18 months (Kemmler et al., 2020b) and who had undergone a seven-month deconditioning period. Recruitment was conducted via an information event by the FrOST study coordinator. In a third step, frail residents of an assisted living facility were recruited. Here, assisted living refers to older people who are still healthy enough to live independently and require only minor assistance in activities of daily living (ADL), e.g., cooking and cleaning. In this setting, participants were addressed via newsletter and the distribution of flyers within the facility. All study prospects who contacted the study centre by phone or E-mail received detailed written study information before signing the informed consent.

### 2.3 Intervention

The WB-EMS equipment (miha bodytec®, Gersthofen, Germany) enables the simultaneous stimulation of an area of about 2,600 cm^2^, innervating calves and thighs, upper arms, buttocks, abdomen, chest, lower and upper back and allowing the separate intensity regulation for each body region. Bipolar electric current with an impulse frequency of 85 Hz, impulse width of 350 µs and a rectangular waveform with a ramp of 0.4 s was applied intermittently with 6 s of stimulation and 4 s of rest (60% duty cycle). This protocol combined easy to perform functional exercises based on the Otago Exercise Programme (Gardner et al., 2001) with the simultaneous electrical stimulation. Safety adjustments to the protocol were based on the experience from previous WB-EMS studies with older adults as well as on the guideline for a safe and effective WB-EMS application (Kemmler et al., 2016b).

All participants completed an eight-week WB-EMS protocol adapted to their functional capacity level (see section below). In total, the training program comprised of ten WB-EMS sessions. After a four-week conditioning period with one training session per week, the frequency was increased to 1.5 sessions per week (3 sessions in 2 weeks) at week five. All training sessions lasted 20 min. In between sessions were three to four rest days for regeneration and adaptation.

Age, frailty and age-related diseases (e.g., diabetes) impair the sensitivity to electrical stimulation, e.g., reduced impulse sensation (Kemp et al., 2014; Palve and Palve, 2018). Therefore, and in contrast to previous WB-EMS studies with older adults, each session started with a five-minute impulse familiarization in a seated or standing position (without simultaneous exercises). The individual rate of perceived exertion (RPE) for each body region was adjusted by feedback of the participants using the Borg CR10 scale (Borg GB, 2010). Because of the high prevalence of cognitive impairment in frail older adults, we combined the Borg CR10 scale with a Faces Pain Scale (Hadjistavropoulos et al., 2014). During the first sessions, the RPE was at a moderate intensity of “somewhat hard” to “hard” (RPE 4–5). After the initial 4 weeks, participants were encouraged to exercise at an RPE of “hard+" to “very hard” (RPE 6–7). The individual stimulation parameters were recorded for each session in order to ensure sufficient progression.

**FIGURE 1:**
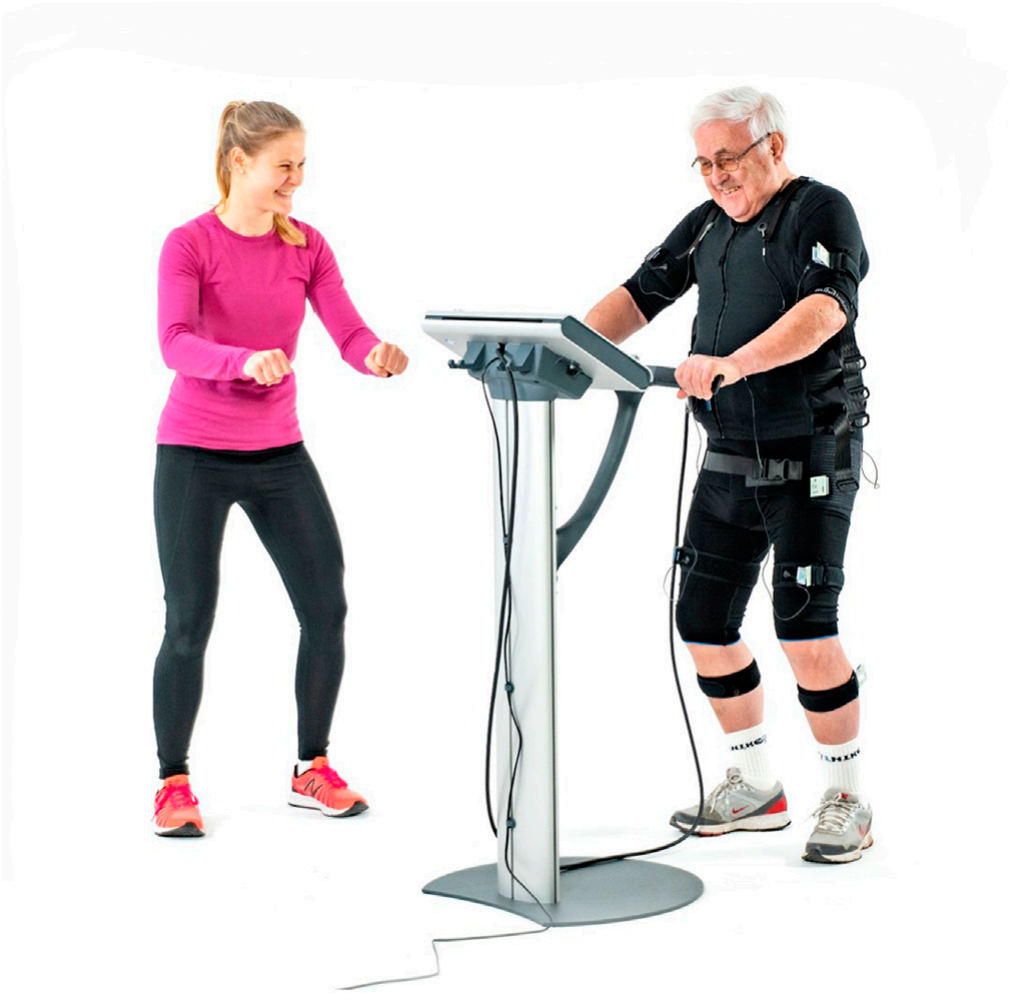
Illustration of WB-EMS.

After the impulse familiarization, the participants performed seven functional exercises for 15 min. These exercises addressed lower extremity strength and balance and differed in their difficulty levels, which were adjusted with regard to time standing (sitting to standing); balance challenge (holding on to not holding on; from larger to smaller base of support, both feet on ground to one-leg stance, open eyes to closed eyes); passive versus active movements (static to dynamic) and functionality (using step board, increasing range of motion, including depth of weight-bearing exercises) (Table 1). All exercises were previously videotaped to provide additional visual guidance to the participants. For each exercise, 12 repetitions were performed with one repetition (3 s concentric—3 s eccentric work) for each stimulation cycle of 6 s. Between each of these seven exercises were pauses of 30 s, in which the stimulation intensity was readjusted by enquiring the current RPE level for each muscle group with subsequent adjustment to the warranted level (RPE 6–7). Licensed EMS trainers instructed the video-guided exercises in 1:1 supervision. Frail individuals used integrated handholds for fall prevention (Figure 1).

**TABLE 1:** Training program by levels of difficulty.

Difficulty level 1 (easy)	Difficulty level 2 (medium)	Difficulty level 3 (hard)
1. Impulse familiarization	1. Impulse familiarization	1. Impulse familiarization
2. Side-by-side stand	2. Semi-tandem/tandem-stand	2. Tandem-stand
3. Calf raises (sitting)	3. Calf raises (standing)	3. Toe walk
4. Toe raises (sitting)	4. Toe raises (standing)	4. Toe raises (standing)
5. Supported sit-to-stands	5. Half squats	5. Squats
6. Small side steps	6. Wide side steps	6. Side lunges
7. Knee extension (sitting)	7. Stepping on stepboard	7. Step-ups on stepboard
8. Backward lunges	8. Backward lunges with added heel raise	8. Back scale

### 2.4 Feasibility

Feasibility was evaluated using:

1) Recruitment rate—ratio of included participants to eligible participants as well as reasons for not participating in the study (El-Kotob and Giangregorio, 2018).
2) Adherence—percentage of training sessions attended by participants (El-Kotob and Giangregorio, 2018).
3) Retention and dropout rate—proportion of participants who remained and dropped out of the study, including reasons for dropouts (El-Kotob and Giangregorio, 2018).
4) Satisfaction was assessed using the Physical Activity Enjoyment Scale for older adults (PACES-8) at weeks 1, 3, and 8 (Mullen et al., 2011). The PACES-8 is an eight-item short version of the Physical Activity Enjoyment Scale (PACES), which has high internal consistency (Cronbach’s *α*= 0.95) in adults with functional limitations (Murrock et al., 2016). Participants rated their enjoyment on a 7-point Likert scale, ranging from for instance “It is very pleasant” to “It is very unpleasant”, with high scores indicating high exercise satisfaction (Mullen et al., 2011). The mean score over all items was calculated.
5) Practicality was assessed by factors addressing the success or failure of execution, the amount/type of resources needed (e.g., time) and the ability of participants to carry out the intervention (Bowen et al., 2009).

WB-EMS was considered feasible for frail older adults living in assisted living facilities, if the 1) recruitment rate was ≥ 50%, 2) adherence was ≥ 80%, 3) retention rate ≥ 80% or dropout rate ≤ 20%, and 4) WB-EMS was rated ≥4 points on the PACES-8 (Harris and Dyson, 2001; Nyman and Victor, 2012; Provencher et al., 2014).

### 2.5 Acceptability

A 20-item questionnaire was used, focussing on key areas of acceptability assessment, e.g., satisfaction, intent to continue use, perceived positive or negative effects (Bowen et al., 2009). The questionnaire consisted of quantitative (dichotomous [yes/no] or 4-point Likert scale) and qualitative (open-ended questions) response formats.

### 2.6 Safety

The incidence of exertional rhabdomyolysis was monitored by the CK activity, a clinically well-validated biochemical marker for the identification of muscle damage (Lippi et al., 2018). CK was measured 15–30 min before, and 48 and 72 h after WB-EMS at weeks 1, 3, and 8. Blood was drawn in a sitting position from an antecubital vein. Past clotting for at least 30 min, serum samples were centrifuged for 15 min at a relative centrifugal force (rcf) of 2,500 g (Centrifuge 5702 R, Eppendorf AG, Germany), and the supernatant was stored frozen at −80°C. After centrifugation all analyses were performed at the Institute of Clinical Chemistry, Laboratory Medicine and Transfusion Medicine, Nuremberg General Hospital, Paracelsus Medical University, Nuremberg, Germany with a Cobas 8,000 with reagents from the manufacturer (Roche Diagnostics GmbH, Mannheim, Germany).

The occurrence of myoglobinuria was assessed by semi-quantitative dipstick urinalysis (Combur^9^ Test®, Roche Diagnostics GmbH, Mannheim, Germany) 24 h after WB-EMS at weeks 1, 3, and 8. Clinically relevant myoglobinuria was defined by myoglobin values of 3+ or 4+ (Schifman and Luevano, 2019). Further, participants were asked before each WB-EMS session, if they observed dark-coloured urine after the last WB-EMS session. In addition, symptoms of rhabdomyolysis (severe muscle pain, muscle weakness) and adverse events were recorded for the previous week before each WB-EMS session (Kim et al., 2016). To assess acute and delayed onset muscle soreness (DOMS), the short form of the Brief Pain Inventory (BPI-SF) was used 24 and 72 h after WB-EMS at weeks 1, 3 and 8 (Radbruch et al., 1999). Participants were instructed to contact their primary care physician and the study physician if adverse reactions occurred.

CK elevations above 5,000 U/l combined with the occurrence of severe muscle pain, muscle weakness, and myoglobinuria were considered as exertional rhabdomyolysis (Veenstra et al., 1994; Fernandes and Davenport, 2019).

### 2.7 Preliminary Efficacy

Preliminary efficacy of WB-EMS was accepted if meaningful improvements in established geriatric tests could be demonstrated. Furthermore, the SPPB was considered responsive to WB-EMS, if a substantial improvement of ≥1.0 points was achieved (Perera et al., 2006).

The following tests were performed by all subjects:

1) Maximum isokinetic hip and knee extension (MIES) and flexion strength (MIFS) were measured bilaterally using an isokinetic leg press (CON-TREX LP, Physiomed, Laipersdorf, Germany). The measurement was performed in a sitting, slightly supine position (15°), supported by hip and chest straps. Range of motion (ROM) was selected between 30° and 90° of the knee angle, with the ankle flexed 90° and the feet firmly fixed with straps positioned on a flexible sliding footplate. Participants were instructed to perform five repetitions with maximum voluntary effort. Assessors motivated participants during the trials. Two trials intermitted by 1 minute of rest were conducted and the better used for analyses.
2) The 30-s Chair-Stand Test (30-STS) and the 5 x Chair Rise (5-STS) measure lower extremity strength and function by rapidly getting up and sitting down from a standard height chair without using the arms. For the 30-STS the maximum number (n) of sit-to-stand movements in 30 s was recorded (Jones et al., 1999). For the 5-STS the time (s) to perform five sit-to-stand movements was taken. Due to a lack of information on psychometric properties, during both tests the young comparison group wore an additional 50% of their body weight (in form of a weight vest and dumbbells) to make them more challenging and potentially more responsive to change.

The following tests were performed by the older participants only:

1) The SPPB, comprising of three components, is a test battery that measures the functional capacity of the lower extremities. Static balance is measured by the ability to hold three positions (side-by-side, semi-tandem, tandem stances) for 10 s. Gait velocity is assessed using the better of two 4 m distance walks at usual pace from a standing position but without the deceleration phase. Functional strength is measured using the 5-STS. Each component is scored from 0 to 4 points. A sum score (0–12 points) is calculated with higher scores indicating better functional capacity (Guralnik et al., 1994). A score of <10 is associated with an increased risk of ADL and mobility-disability as well as all-cause mortality (Guralnik et al., 2000; Pavasini et al., 2016).
2) The Timed Up-and-Go Test (TUG) measures functional mobility in older people by the time (s) required to stand up from a chair, walk 3 m at the usual pace without walking aids if possible, turn around, return and sit down again (Podsiadlo and Richardson, 1991).
3) The Choice Stepping Reaction Time Test (CSRT) was used to measure response selection and reaction time. The CSRT is associated with balance control, lower extremity strength, and falls in older adults (Lord and Fitzpatrick, 2001; Delbaere et al., 2016). A simple “low-tech” CSRT was used (Delbaere et al., 2016). Following eight practice trials, the time (s) taken for a sequence of 12 steps was measured. Participants were required to step as fast as possible into one of four step directions (side-right, side-left, front-right, front-left) following verbal instructions.
4) The maximum handgrip strength (kg) of the dominant hand was tested as the average of three trials using a JAMAR hand dynamometer (JAMAR® PLUS+, Performance Health Supply, Cedarburg (WI), United States). The grip width was adjusted to the participants hand size. Subjects were seated in an upright sitting position with the arm bent at 90° and instructed to squeeze with maximum force. Assessors motivated participants during the trials.

Additionally, diseases and medications were assessed by questionnaire and physician’s letters. The cognitive status was evaluated by the Mini Mental State Examination (MMSE) (Folstein et al., 1975). Anthropometric markers (e.g., height, weight, body mass index (BMI), skeletal muscle mass, body fat mass, percent body fat) were measured using bioelectrical impedance analysis (InBody 230, Biospace Co., Korea) under fasting condition in the morning.

### 2.8 Statistical Methods

Due to the small sample size, nonparametric methods were applied (Kitchen, 2009; Van Buren and Herring, 2020).

CK kinetics within-group were analysed using Friedman-ANOVAs for repeated measurements. Bivariate correlations between muscle pain and CK elevations were examined using Spearman’s rank correlation. For efficacy outcomes, change scores were calculated and the Wilcoxon signed-rank test was used for within-group differences. All differences between-groups were investigated using the Mann-Whitney U-test and the Kruskal-Wallis test for continuous variables and the Pearson’s chi-squared test for categorical variables. Significance was accepted at *p* < 0.05. For post-hoc tests in repeated measure analyses, the significance level was adjusted using the Bonferroni correction (Pereira et al., 2015).

The effect size r (weak 0.1, medium 0.3, strong 0.5 (Cohen, 1988)) was calculated as follows (Rosenthal, 1991):

$$r=\;\left|\frac{z-score}{\sqrt{number\;\left(N\right)\;of\;total\;obeservations}}\right|$$

Feasibility data was analysed descriptively. Qualitative items of the self-developed questionnaire were first summarized and categorized by content analysis and then evaluated descriptively.

We used the IBM Statistical Package for the Social Sciences (SPSS, version 26) for statistical analysis.

## 3 Results

**FIGURE 2:**
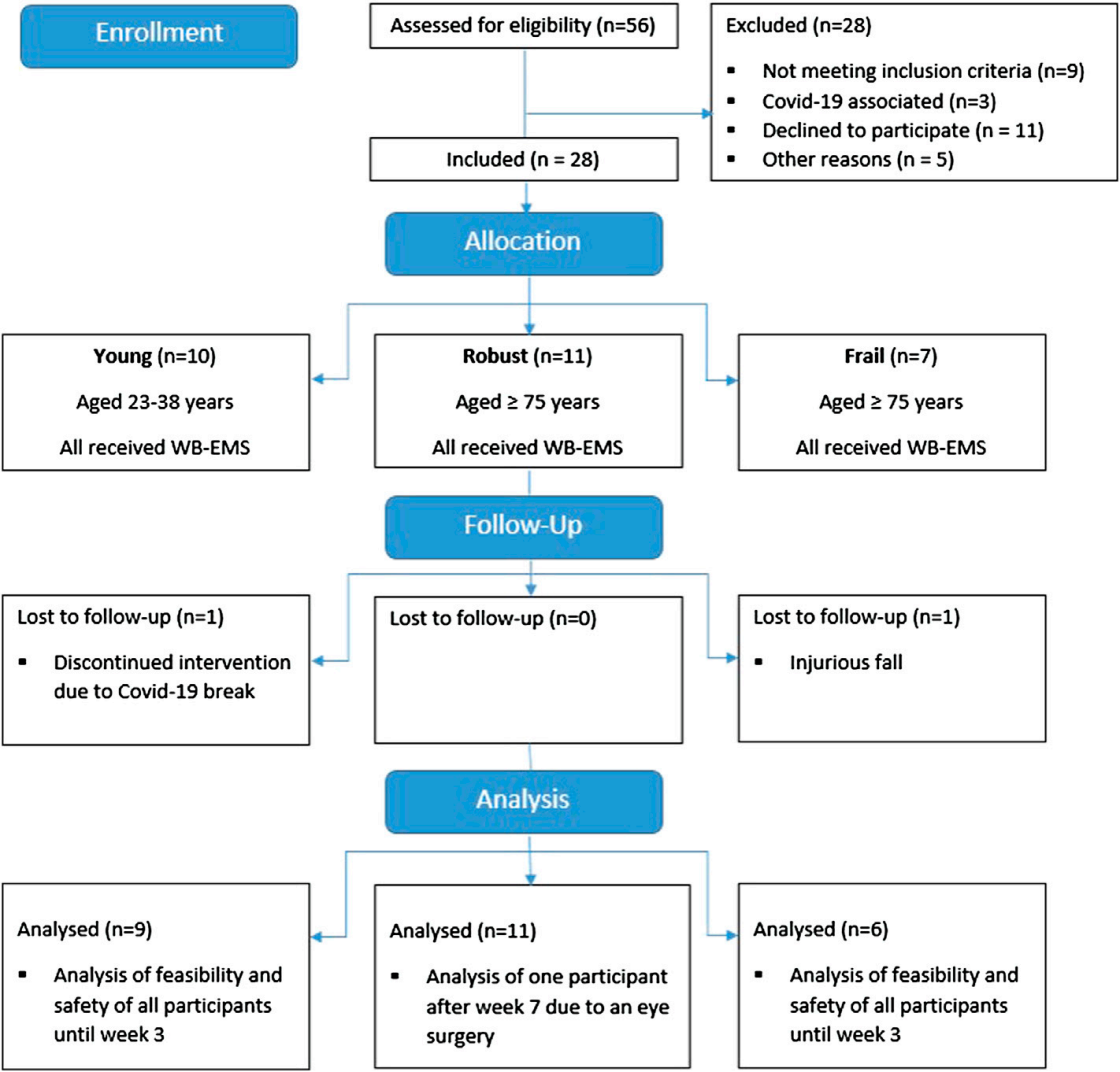
Diagram of participant flow.

Eighteen older (11 robust (RG), 7 frail (FG)) and ten young subjects (YG) were included. The participant flow is displayed in Figure 2. Firstly, the YG was recruited in February 2020 and started the intervention in March 2020. Due to the outbreak of the Covid-19 pandemic, the intervention stopped after three sessions and was restarted in July 2020. One participant dropped out before the re-start due to time reasons. The nine remaining subjects completed the eight-week intervention and performed at least nine of ten WB-EMS sessions. Secondly, the RG was recruited. With the exception of one participant who finished 1 week early due to elective eye surgery, the robust participants completed all WB-EMS sessions. Thirdly, the FG as well as two additional robust subjects were recruited in the assisted living facility. All but one participant, who had to terminate early after three training sessions due to an injurious fall unrelated to the intervention, completed all WB-EMS sessions.

At baseline, the YG and the older participants differed significantly for most characteristics. The RG and FG demonstrated significant differences in gender proportion, number of diseases, percent body fat as well as maximum leg strength (MIES, MIFS), while skeletal muscle mass, body fat mass, 30-STS, 5-STS were borderline non-significant (Table 2).

**TABLE 2:** Baseline Characteristics of the three study groups.

	YG (*n* = 10)	RG (*n* = 11)	FG (*n* = 7)	*p*
Age (year)	29.1 ± 6.4	79.5 ± 3.6	81.3 ± 3.5	<0.001^a^
				0.246^b^
Gender (male/female)	3/7	9/2	2/5	0.025^c^
				0.024^d^
Number of medications (n)	0.8 ± 0.6	4.2 ± 3.7	5.1 ± 5.6	<0.001^a^
				0.328^b^
Number of diseases (n)	0.2 ± 0.4	3.2 ± 1.9	5.6 ± 2.3	0.002^a^
				0.044^b^
Mini Mental Status (MMSE)	N/A	28.3 ± 1.8	28.3 ± 1.3	-
				0.860^b^
BMI (kg/m^2^)	22.2 ± 2.7	24.4 ± 2.3	26.2 ± 3.8	0.043^a^
				0.328^b^
Skeletal muscle mass^e^ (kg)	29.2 ± 8.9	26.9 ± 3.0	24.0 ± 2.5	0.187^a^
				0.056^b^
Body fat mass^e^ (kg)	13.4 ± 3.7	20.3 ± 4.3	26.0 ± 7.4	0.001^a^
				0.085^b^
Percent body fat^e^ (%)	20.9 ± 5.1	28.9 ± 4.6	36.3 ± 6.7	<0.001^a^
				0.015^b^
Creatinkinase (U/l)	103.9 ± 66.7	107.7 ± 34.2	85.7 ± 58.7	0.464^a^
				0.126^b^
MIES (N)	3,497 ± 952	2,005 ± 647	970 ± 300	<0.001^a^
				0.001^b^
MIFS (N)	1,669 ± 501	766 ± 368	278 ± 87	<0.001^a^
				0.002^b^
30-STS (n)	16.4 ± 2.8^f^	14.6 ± 3.1	11.6 ± 3.7	<0.001^a^
				0.085^b^
5-STS (s)	8.8 ± 1.3^f^	10.8 ± 2.0	14.0 ± 5.8	0.012^a^
				0.085^b^

Values are presented as mean ± standard deviation.

_a_ Differences between YG, RG, and FG, analysed by Kruskal-Wallis-Test.

b Differences between RG and FG, analysed by Mann-Whitney U-test.

c Differences between YG, RG, and FG, analysed by Pearson’s chi-squared test.

d Difference between RG and FG, analysed by Pearson’s chi-squared test.

e Assessed by bioelectrical impedance analysis (InBody 230).

f Wore additional 50% of their body weight.

YG, young group; RG, robust group; FG, frail group; N/A, not applicable; MMSE, mini mental status examination; BMI, body mass index; MIES, maximum isokinetic hip/knee extension strength; MIFS, maximum isokinetic hip/knee flexion strength; 30-STS, 30-s Chair-Stand Test; 5-STS, 5 x Chair Rise.

### 3.1 Feasibility

The YG and RG demonstrated higher recruitment (66.7 and 64.6%), adherence (92.0 and 97.3%), and retention rates (90.0 and 90.9%) and lower dropout rates (10.0 and 9.1%) than the FG in the assisted living facility, in which the recruitment rate (46.2%) was lower. The FG completed 53 of the total 60 WB-EMS sessions (88.3%), had a retention rate of 83.3% and a dropout rate of 16.7%.

Reasons for non-inclusion of frail older individuals were pacemakers, CKD stage 4, acute fracture, severe polyneuropathy, uncontrolled hypertension, age <75 years, fear of electricity, and unwillingness to participate for unspecified reasons. In addition, several potentially eligible individuals declined to participate due to the Covid-19 circumstances.

All participants were able to complete the training protocol. In particular, frail individuals were able to stand independently for 20 min without having to sit down during the sessions. All participants of the FG started with *difficulty level 2*. Two participants of the FG were able to improve to *difficulty level 3* as the training progressed, while none regressed to a lower level. The RG and YG exercised at *difficulty level 3*. The five-minute impulse familiarization was long enough to adjust the stimulation intensity for each body region individually. The 30 s between-exercise-pauses were adequate to sufficiently readjust and progress the stimulation intensity during the training. After 4 weeks, all participants were able to reach an RPE of “hard+” to “very hard” (RPE 6–7). Furthermore, all participants were able to follow the video-guided instructions, although the FG required more additional verbal and tactile/haptic feedback by instructors.

While the training’s duration of 20 min was the same for all groups, the FG required more time (≈20 min) than the YG and RG (≈10 min) due to a greater need for assistance in dressing and undressing. Age- or disease-associated factors such as changes in body composition, such as height loss, scoliosis, obesity, or cachexia, influenced the accurate fitting of the electrodes and electrode vests. For example, in obese individuals, special inserts were required. In incontinent participants, electrode pads were used that could be fixed inside of the adult diaper, allowing the stimulation of the gluteal muscles.

All groups rated WB-EMS on average > 4 on the PACES-8 (FG with 4.8 ± 0.3; RG with 5.0 ± 0.7; YG with 5.2 ± 1.1). There was no significant between-group difference in training satisfaction (*p* = 0.680).

Acceptability of WB-EMS was overall rated as “very good” (33%) or “good” (67%) in the FG (*n* = 6). Of the RG (*n* = 11), 27% rated WB-EMS as “very good,” 64% rated it as “good,” and one person rated it as “not as good”. The majority (67%) of the FG would continue to exercise with WB-EMS. Subjects with impaired lower extremity function (SPPB <10) seemed to prefer WB-EMS to “conventional exercise” rather than functionally unimpaired individuals (67 vs. 9%). Reasons provided by the FG included (frequencies in parentheses): subjective effectiveness (50%), exercise program characteristics (50%), personalized training approach, interesting/innovative training method, time efficiency, reduced feeling of exhaustion, and fun (single reports). The humidity/coldness (83%) caused by the moistening of the EMS electrodes, the time-consuming dressing and undressing of the equipment and the feeling of shame due to wearing the tight-fitting EMS functional underwear (single reports), were rated as negative. More than half (59%) of all older subjects reported subjective improvements in their health or functional capacity. Perceived effects of WB-EMS were improvements in pain (24%), mobility (24%), vitality and well-being (24%), gait and stability (18%), strength (12%), as well as better continence and sleep (single reports).

### 3.2 Safety

**FIGURE 3:**
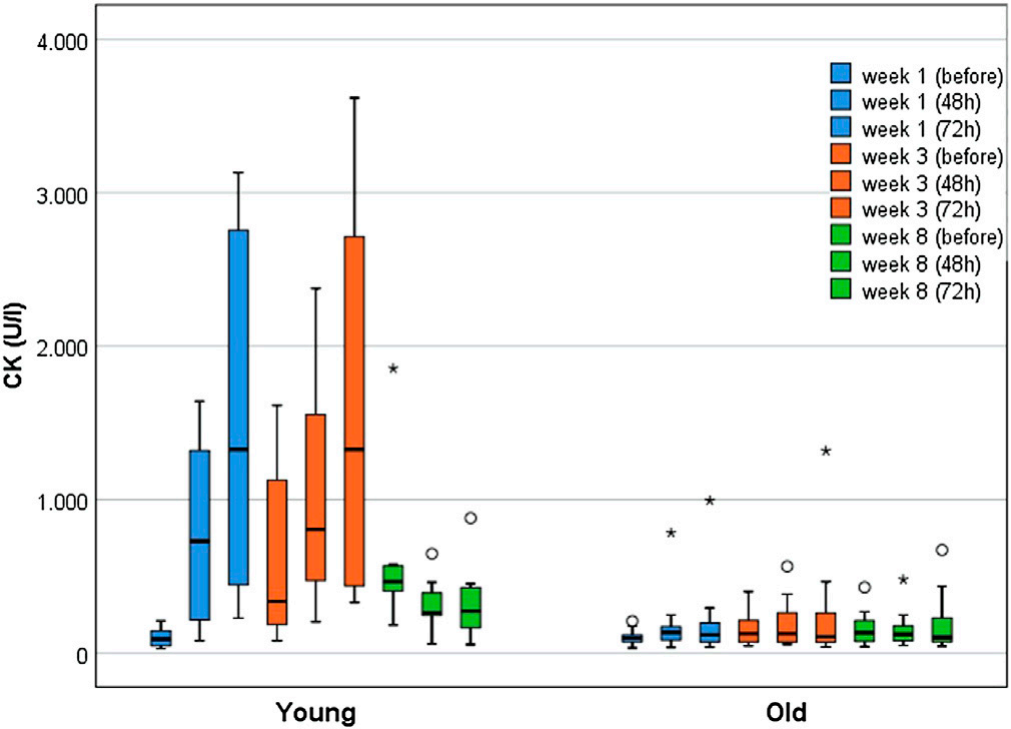
Boxplots displaying the median with interquartile range for CK kinetics of young (*n* = 10) and older participants (*n* = 18) over the course of 8 weeks (immediately before, after 48 h, after 72 h) WB-EMS. Starting from week 5, three WB-EMS sessions were performed within 2 weeks with breaks of 4 days. Therefore, CK elevations before the last training in week 8 represent the 96 h CK peak of the previous WB-EMS session.

Figure 3 illustrates CK kinetics at weeks 1, 3, and 8 immediately before, 48 and 72 h after WB-EMS separated by age groups. Of note, the CK elevations before the last training in week 8 represent the 96 h CK peak of the previous WB-EMS session.

At baseline, the YG (*n* = 10; Mdn_young_ = 90 U/l [IQR 46–159]) and the older participants (*n* = 18; Mdn_old_ = 96 U/l [IQR 68–122]) did not differ (*p* = 0.832). The YG demonstrated significant and substantially greater CK activity over the course of the training (*p* < 0.001). In contrast, the older groups did not show significant changes (*p* = 0.213). The most pronounced between-group difference in CK activity was observed 72 h after the first training (Mdn_young_ = 1,326 U/l [IQR 396–2,806] vs. Mdn_old_ = 118 U/l [IQR 69–197]; *p* < 0.001; r = 0.75). In the YG, the highest CK elevations (relative to baseline) were found 72 h after the WB-EMS session in week one and three, with increases by the factor 14.6 (Mdn_young_ = 1,326 U/l [IQR 396–2,806]; *p* < 0.001; r = 1.0) and of 15.5 (Mdn_young_ = 1,327 U/l [IQR 434–2,737]; *p* < 0.001; r = 1.08) respectively. The older participants showed the highest CK increase (1.8-fold (Mdn_old_ = 126.5 U/l [IQR 70–263]; *p* = 0.016; r = 0.50) 48 h after the third WB-EMS session.

Examining the RG and FG separately, the RG showed a significant 3.0-fold CK increase until week 3 (*p* = 0.010), whereas the FG demonstrated only borderline significant changes by the factor 1.5 during the first week (*p* = 0.05), peaking at 72 h after WB-EMS (*p* = 0.048; r = 0.64). Both groups did not differ significantly at baseline (Mdn_frail_ = 74 U/l [IQR 52–105]; Mdn_robust_ = 112 U/l [IQR 79–135]; *p* = 0.126). The largest between-group difference in CK activity was found 72 h after the third training (Mdn_robust_ = 227 U/l [IQR 104–417] vs. Mdn_frail_ = 70 U/l [IQR 54–106]; *p* = 0.015; r = 0.56).

None of the subjects exceeded the *a priori* defined threshold of ≥5,000 U/l for severe rhabdomyolysis. Clinical signs of rhabdomyolysis (severe muscle pain and weakness, myoglobinuria) were neither observed nor reported. Occasionally, more severe muscle pain was reported, but was interpreted as normal exercise-related muscle soreness by the participants. In weeks 1 and 3, 72 h after WB-EMS, in which the CK activity was most pronounced, there was a low-moderate but non-significant positive correlation between muscle pain and CK elevations for the YG (week 1: r_s_ = 0.578; week 3: r_s_ = 0.22). In older individuals a very low and non-significant, negative correlation was found (week 1: r_s_ = −0.174; week 3: r_s_ = −0.03). Myoglobinuria was neither self-reported nor did it occur in the semi-quantitative dipstick urinalyses. Occasionally, nonspecific symptoms, such as malaise or nausea, were reported, but judged as not WB-EMS-related by the study physician.

### 3.3 Harms

After the third WB-EMS session, one participant of the RG expressed slight pain and sensory complaints on the elbow. Due to a known case of Sudeck’s disease, this participant did not undergo stimulation of the arms in the further course of the intervention.

Three subjects (*n* = 1 RG, *n* = 2 FG) reported one fall at home. Two of the three subjects suffered no or only minor injuries (sprained thumb). One frail individual suffered severe injuries requiring medical treatment (ulnar fracture, compartment syndrome, laceration) as a result of a fall down stairs, which required hospitalization and resulted in exclusion from the study. All falls were judged unrelated to the WB-EMS protocol.

### 3.4 Preliminary Efficacy

The three groups differed significantly at baseline (Table 2). The YG improved significantly in MIES (10.4 ± 12.8%; *p* = 0.038; r = 0.49), 30-STS (24.3 ± 12.2%; *p* = 0.007; r = 0.64), and 5-STS (22.3 ± 8.0%; *p* = 0.008; r = 0.63). The improvement by 6.7% in MIFS was not significant (*p* = 0.11). The RG significantly improved in MIFS (35.2 ± 33.3%; *p* = 0.008; r = 0.57), but not in MIES (5.3 ± 13.6%; *p* = 0.155). The RG further showed significant improvements in 30-STS (23.9 ± 11.6%; *p* = 0.003; r = 0.63) and 5-STS (17.7 ± 14.6%; *p* = 0.008; r = 0.57). In the FG significant improvements were found for MIES (29.1 ± 33.5%; *p* = 0.043; r = 0.64), MIFS (81.3 ± 60.7%; *p* = 0.043; r = 0.64), 30-STS (17.6 ± 12.1%; *p* = 0.043; r = 0.58) and 5-STS (18.0 ± 25.8%; *p* = 0.046, r = 0.58). There were no significant between-group differences with regard to changes in MIES (*p* = 0.479), MIFS (*p* = 0.527), 5-STS (*p* = 0.601) and 30-STS (*p* = 0.114) after 8 weeks of WB-EMS.

**FIGURE 4:**
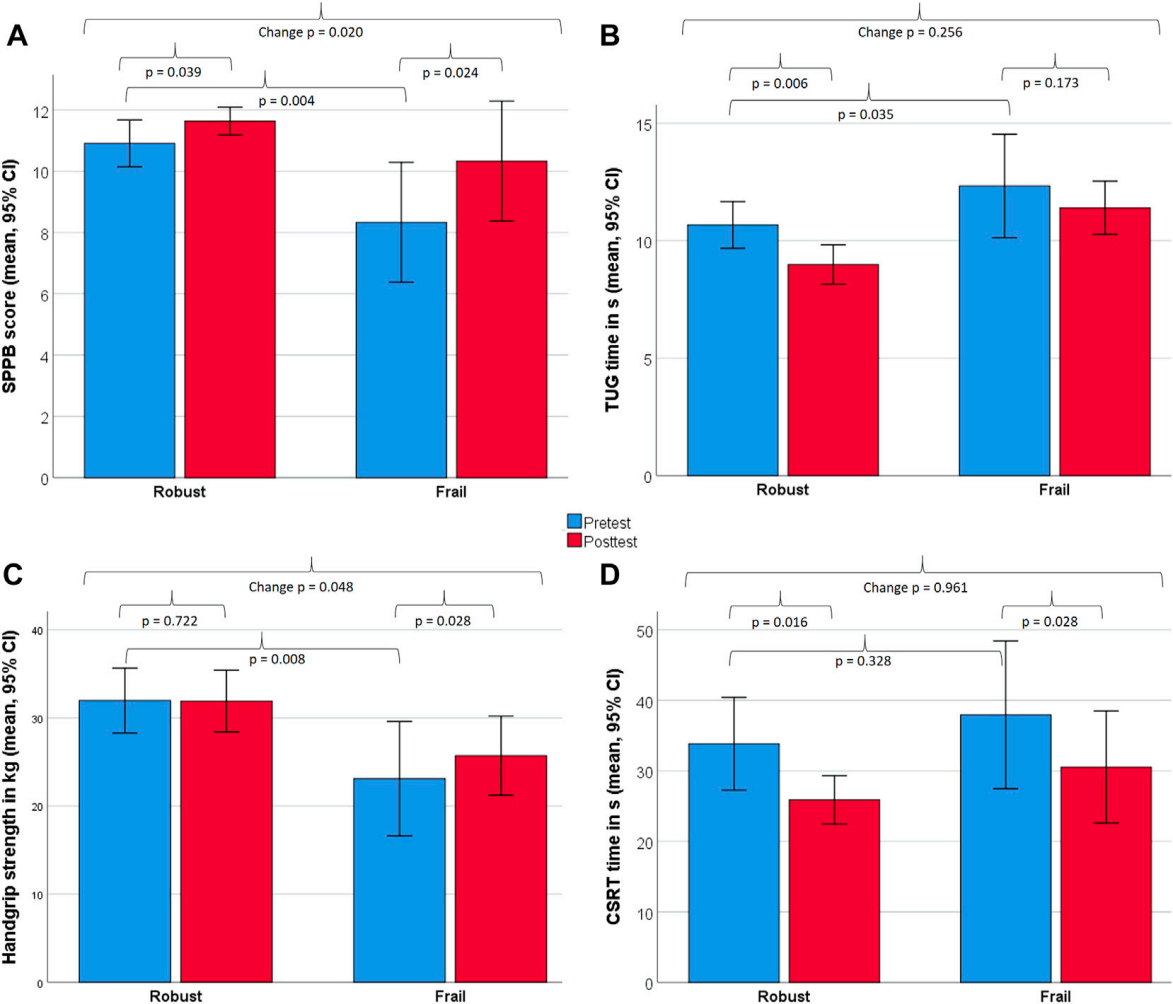
Changes between baseline and 8 weeks in strength and functional capacity measures for robust and frail individuals. Displayed are within- and between-group differences for the Short Physical Performance Battery [SPPB, **(A)**], Timed-up & Go test [TUG, **(B)**], handgrip strength **(C)**, Choice Stepping Reaction Time [CSRT, **(D)**]. All measures were analysed non-parametrically using the Wilcoxon signed-rank test (within-group) and the Mann–Whitney U-test (between-group).

Figure 4 illustrates further changes in strength and functional capacity, assessed in the FG and RG. The FG improved significantly in the SPPB (2.0 ± 0.6 points; *p* = 0.024; r = 0.65), in handgrip strength (11.3 ± 10.8%; *p* = 0.028; r = 0.64) and the CSRT (19.5 ± 10.4%; *p* = 0.028; r = 0,64), but not the TUG (*p* = 0.173). The RG improved signficantly in the SPPB (0.7 ± 1.0 points; *p* = 0.039; r = 0.44), the CSRT (23.5 ± 16.4%; *p* = 0.016; r = 0.51) and the TUG (15.8 ± 10.7%; *p* = 0.006; r = 0.59), but not in handgrip strength (*p* = 0.722). Significant between-group differences in change scores in favor for the FG were found for SPPB (*p* = 0.02) and handgrip strength (*p* = 0.048), but not for the TUG (*p* = 0.256) and CSRT (*p* = 0.961).

## 4 Discussion

This pilot study aimed to evaluate the feasibility and safety of an eight-week WB-EMS program in frail older people living in assisted living facilities. By showing preliminary efficacy of WB-EMS, the objective was to demonstrate that observed safety did not cause a lack of efficacy.

The results demonstrate the feasibility of WB-EMS in frail individuals residing in assisted living facilities, although we just missed a recruitment rate of 50%. However, adherence, retention and dropout rates were above the *a priori* defined thresholds. All groups indicated high training satisfaction with WB-EMS. The intervention also proved to be safe in respect to the risk of rhabdomyolysis. We observed CK elevations below the *a priori* defined threshold of ≥ 5,000 U/l. Furthermore, no typical clinical signs of exertional rhabdomyolysis (severe muscle pain and weakness, myoglobinuria) were observed (Kim et al., 2016). Safety adjustments, such as the four-week conditioning phase, did not lead to a lack of efficacy. Except for the TUG, the FG improved significantly in all strength and functional capacity outcomes. Furthermore, the FG showed the greatest magnitude of change in maximum leg strength (MIES and MIFS) in comparison to the RG and YG and significantly greater changes than the RG in handgrip strength and the SPPB, indicating high trainability.

### 4.1 Feasibility

In comparison to recruitment rates of 70% in exercise interventions seen in community-dwelling older adults, the recruitment rate of 46% was lower among the FG in the assisted living facility (Nyman and Victor, 2012). This may have been due to a general restraint because of the fear of Covid-19 infections. In fact, several potential participants apologised for non-participating due to this reason. Additionally, factors, such as poor health, mobility problems, chronic diseases, fatigue, and cognitive impairment, which affect an individual’s ability to consent to and participate in interventional studies as well as distrust and fear of strangers may account for lower recruitment rates in frail individuals (Harris and Dyson, 2001; Provencher et al., 2014). On the other hand, only mildly frail older adults often do not self-identify as “frail” and therefore may not respond to recruitment. Hence, research experience indicates recruitment rates around 50% among frail cohorts (Harris and Dyson, 2001).

Furthermore, age and the level of disability affects adherence and retention rates of older people (Kempen and Vansonderen, 2002). Due to a high vulnerability, frail individuals may have more difficulties dealing with side effects of exercise interventions (e.g., muscle soreness) and are at higher risk of adverse events (Provencher et al., 2014). Surprisingly, the adherence of 88% and retention rate of 83% in our study was quite high in this population (Provencher et al., 2014). In comparison to previous WB-EMS studies with older non-frail people, the adherence was comparable; however the dropout rate was higher (17 vs. 10%) (Kemmler et al., 2016a; Kemmler et al., 2017).

As no monetary or other incentives were provided, the relatively high adherence and retention is likely due to high training satisfaction, perceived effectiveness, and the “personal training” approach of WB-EMS. Studies show, that the lack of perceived benefits, distrust of research staff and mobility problems are among the key barriers for intervention participation in frail individuals (Provencher et al., 2014). Therefore, studies with supervised programs and a high frequency of interpersonal contacts have higher adherence rates (Picorelli et al., 2014).

In addition, the participants’ belief that WB-EMS was beneficial to their health may have supported adherence and retention. Studies show that anticipated ADL improvements are a strong intrapersonal motivator for exercise in older people (Dedeyne et al., 2018). Accordingly, 59% of older study participants in the current study indicated that their health had improved. In addition, the majority of the physically impaired participants (SPPB < 10) intended to continue with WB-EMS, whereas the more robust preferred other exercise modalities. For older people with functional limitations, the personal training approach of WB-EMS could therefore be a preferable training form. However, WB-EMS is a rather expensive personal training approach. It is likely, that recruitment would have been more challenging and long-term adoption would be lower if participants had to pay for the training.

### 4.2 Safety

To the best of our knowledge, this is the first study conducting a rather close meshed kinetic over several weeks in older, including frail, individuals during a WB-EMS training cycle.

CK activity was lower in all groups than has been previously reported in WB-EMS studies in younger and trained individuals with more strenuous training protocols (Teschler et al., 2016; Hettchen et al., 2019) and below the threshold of 5,000 U/l, which is associated with a higher risk of acute kidney injury (Brown et al., 2004; Rodríguez et al., 2013) and therefore proposed as a pragmatic cut-off for exertional rhabdomyolysis (Fernandes and Davenport, 2019). However, according to classifications grading rhabdomyolysis into mild (<10 times the upper limit of the norm for men [<190 U/l] and women [<170 U/l]), moderate (10–49 times of the norm) and severe (≥50 times of the norm) (Thomas et al., 2005; Kim et al., 2016), some individuals in the YG but not the RG and FG showed values inside the mild-to-moderate range. Nevertheless, a high CK increase after eccentric strength training is common (Fernandes and Davenport, 2019). For this reason, the combination of myopathic symptoms, such as severe muscle soreness, weakness and myoglobinuria, and substantial CK elevations (>50,000 U/l) are indicative of exertional rhabdomyolysis, while CK alone may be sensitive but not specific (Kim et al., 2016; Fernandes and Davenport, 2019). Therefore the YG showed a physiological CK response within the normal range of an eccentric strength training (Baird et al., 2012). Furthermore, in week 8, the YG showed a noticeable repeated bout effect, which represents a muscular adaptation to WB-EMS (Nosaka and Aoki, 2011).

In addition, substantial age differences were observed. The RG showed significantly lower maximum CK elevations than the YG (1,317 U/l vs. 3,617 U/l), while the FG demonstrated only slight CK elevations above the clinical norm for men and women (Thomas et al., 2005). Despite the fact that after a four-week conditioning period all groups trained with an RPE of 6-7, the magnitude of between-group differences in CK activity is surprising. Physical capacity and cognitive function may have affected perceived exertion and resulted in a lower than intended RPE, although studies show no significant difference between young and older adults (Groslambert and Mahon, 2006). On the contrary, older individuals tend to underestimate RPE due to a lack of “conscious muscle contractility awareness” (Pincivero, 2011; Morishita et al., 2019). Nevertheless, Borg Scale measurements have proven to be valid rating methods of RPE in frail older adults (Mendelsohn et al., 2008) as well as in patients with neuromuscular impairments, such as Parkinson’s disease (Penko et al., 2017).

Age differences in CK activity appear to be related to the proportion of type II muscle fibres, which are more susceptible to damage after eccentric exercise (Koch et al., 2014). Because age-related muscle loss is particularly associated with loss of type II fibres (Lang et al., 2010), older people appear to be less susceptible to exercise-induced muscle damage (Koch et al., 2014). This further suggests that, because the loss of type II fibres in the sarcopenic process is more advanced in frail individuals (Miljkovic et al., 2015), the FG has lower CK elevations in comparison to the RG. Consistent with our findings, other WB-EMS studies that analysed CK before and after several months of WB-EMS (8–16 weeks, 1.5–3 x 20–40 min/week) showed similar CK activity in older people (Kemmler et al., 2020c; Kim and Jee, 2020).

In addition, CK activity is associated with the mechanical and metabolic stress to which muscles are exposed during training (Baird et al., 2012; Koch et al., 2014). Moreover, the speed at which muscle contractions are performed seems to have an influence on the magnitude of CK elevations. For instance, fast (210°/s) isokinetic elbow flexions cause an about 4.5-fold higher CK activity than slow movements (30°/s) (Chapman et al., 2006). Although we attempted to standardize our training protocol, there were slight differences in the execution velocity of some exercises (e.g., lunges) due to functional limitations in the FG.

Furthermore, immobilization and low physical activity after eccentric exercise results in blunted CK activity (Sayers et al., 2000). This could be an explanation for the more pronounced CK elevations in several robust subjects. The RG were generally more active than the FG and additionally engaged in activities such as cycling or gardening. The individual with the highest CK elevations (1,317 U/l) of all older participants was a master athlete. Simultaneously to WB-EMS, he carried out several high-intensity running and cycling activities each week.

Sex differences (82% of the RG were male, 71% of the FG female) could have also caused differences in CK activity. For example, women may be less susceptible to exercise-induced muscle damage (Koch et al., 2014). However, the available data is contradictory. Because women tend to be smaller than men, surface electrodes cover a relatively larger muscle area and therefore activate more motor units by stimulation in females than males (Kern et al., 2014). Additionally, due to higher sensory and supramotor excitability, women seem to be more susceptible to surface electrical stimulation (Maffiuletti et al., 2008). Both should lead to a higher muscle activation and more pronounced CK activity in women than in men.

### 4.3 Preliminary Efficacy

All groups were able to improve in most strength and functional capacity outcomes with large effect sizes between 0.49 and 0.64, with the FG demonstrating the greatest effect sizes for the SPPB (0.65), handgrip strength (0.64), MIES (0.64), MIFS (0.64), and the CSRT (0.64). The overall magnitude of the improvements indicate a sufficient stimulation and training intensity.

The FG was able to improve in all strength and functional capacity outcomes, with the exception of the TUG. Although the improvement in the TUG was not significant, the total change was similar to another trial (7.6% vs. 8,3%), in which EMS was applied to lower extremity muscles with a comparable intervention duration of 8 weeks (Cvecka et al., 2015). This indicates that significant improvements might be expected with a larger sample size.

The SPPB improvement by two points in the FG was substantial and clinically relevant (Perera et al., 2006). Five of six individuals with a baseline SPPB score of 9 points or lower were above the clinical threshold of 10 points, which indicates robustness (Cesari et al., 2017). Furthermore, the SPPB demonstrated sufficient responsiveness to WB-EMS, even in the RG, which showed relatively high baseline values (Perera et al., 2006).

The increase of 11.3% in handgrip strength was more pronounced than in former WB-EMS studies in older women and men ≥ 70 years with considerably lengthier intervention periods (5.6–10.5% in 16–54 weeks) (Kemmler et al., 2017; von Stengel et al., 2015). Handgrip strength is considered a surrogate for overall muscle strength, especially of the upper limbs (Beaudart et al., 2019; Landi et al., 2020), as well as a prognostic marker for all-cause and disease-specific mortality and morbidity, functional capacity, bone mineral density, falls, fractures, cognition and depression, problems associated with hospitalization, and loss of independence (Norman et al., 2011; Bohannon, 2019). However, studies are required to demonstrate that improvements in handgrip strength are directly associated with improvements in these important geriatric outcomes.

Additionally, lower extremity strength improved in all participants. The RG and FG combined achieved a significant increase of 9.8% in MIES. This change is comparable to improvements seen in other WB-EMS studies in older people but with lengthier intervention periods (9.5–9.8% in 14–54 weeks) (Kemmler et al., 2010; Kemmler et al., 2012; Kemmler et al., 2018). With regard to the FG, improvements in MIES (29.1%) and MIFS (81.3%) were substantially greater than in previous WB-EMS studies. This is in contrast to a secondary analysis of data from WB-EMS trials, that investigated the trainability of leg strength over the lifespan (27–89 years) in men aged 27–89 years after 14–16 weeks of WB-EMS (von Stengel and Kemmler, 2018). While in this analysis the trainability of MIES (but not MIFS) tended to decrease with older age (*p* = 0.06), our FG showed the relative greatest improvements in maximum leg strength of all participants. Due to their generally lower functional capacity, frail older people may have greater potential for improvements. Accordingly, other studies, which also applied a HIT protocol in frail individuals demonstrated similar results to our findings for MIES (29.1%) (Seynnes et al., 2004; Sahin et al., 2018). For instance, one RCT with 48 physically frail subjects (85.0 ± 5.5 years), 16 of whom performed a HIT over an eight-week period (3x/week) improved significantly by 31.7% in knee extensor strength, while the passive control group deteriorated (Sahin et al., 2018). This underscores not only the trainability and great potential for improvement for frail, older individuals but also the time efficiency of WB-EMS.

Besides these improvements in strength and functional capacity, the 20% improvement in CSRT might also indicate a reduced fall-risk in older participants (Schoene et al., 2017; Okubo et al., 2021). This large change is of interest, as movements during WB-EMS were performed slowly. However, the recruitment of type II fibres may have enhanced the ability to react quickly.

### 4.4 Limitations

We acknowledge several limitations of this study. First, due to the small sample size, that is common to feasibility studies, our findings cannot be generalised. Second, there was an unequal gender distribution in all groups. This could have influenced the response to WB-EMS, e.g., the strength gain or CK activity (Koch et al., 2014). Third, the TFI as frailty criterion is a bio-psycho-social frailty instrument. As such, it was affected by non-physical components, such as the Covid-19 related restrictions. Not all of our participants (3 of 7) were classified as frail based on physical components of frailty alone. Hence, studies are needed that investigate the relative efficacy of WB-EMS on sub-components of frailty in a bio-psycho-social context or, considering the large physical frailty component, include participants based on a physical frailty index, such as the Fried phenotype (Fried et al., 2001). However, study results show that the Fried phenotype and multidimensional operationalizations measure partly the same construct (Van der Elst et al., 2019) and there appears to be a linear relationship, with robust individuals consistently obtaining better results compared to those classified as frail in psychological and social factors (Op het Veld et al., 2015). Fourth, the Covid-19 pandemic and associated restrictions affected recruitment, making it particularly challenging to recruit frail, older individuals. One frequently mentioned cause for non-participation was fear of contracting Covid-19. Our recruitment rate was thus influenced by this circumstance. Fifth, several WB-EMS specific contraindications, such as pacemakers and CKD stage 4 or higher led to the exclusion of several participants fulfilling the frailty criteria, making WB-EMS currently unsuitable for high risk populations such as nursing home residents. Future studies need to clarify if all contraindications are justified. Sixth, WB-EMS is a resource intense intervention caused by the individual supervision, which makes it harder to be implemented. However, this also enables a safe training in frail individuals and in combination with the relatively large preliminary treatment effects potentially makes WB-EMS especially suitable for participants with greater impairments that may be less suitable for group interventions or in need for personal training. In addition, the short duration and low training frequency required make it suitable for individuals who are not motivated to participate in frequent group exercise programs. Finally, the relative contributions of WB-EMS and functional strength and balance training remains to be determined in future studies.

## 5 Conclusion

WB-EMS appears to be feasible, safe, and demonstrated preliminary efficacy with large effect sizes in frail, older adults. Especially for older people with functional limitations, the personalized WB-EMS approach could be a suitable training paradigm. Future studies need to further evaluate efficacy of WB-EMS on functional capacity and other relevant geriatric outcomes in adequately powered RCTs. In addition, it is required to investigate the relative effects of WB-EMS and of functional exercises.

## Data Availability

The datasets generated and/or analysed during the current study are available from the corresponding author upon reasonable request.
